# Yield Stability and Adaptability of Black Soybean Line BSCM41 Support Bi-Directional Seed Rotation System in Northeast Thailand

**DOI:** 10.3390/plants15182820

**Published:** 2026-09-14

**Authors:** Kitti Phosuk, Jariya Chinnarat, Tidarat Monkham, Jirawat Sanitchon, Jamnan Khodphuwiang, Suntit Reewarabundit, Sompong Chankeaw

**Affiliations:** 1 Department of Agronomy, Faculty of Agriculture, Khon Kaen University, Khon Kaen 40002, Thailand; kittiph@kkumail.com (K.P.); jariyachinnarat@kkumail.com (J.C.); tidamo@kku.ac.th (T.M.); jirawat@kku.ac.th (J.S.); 2 Plant Breeding Research Center for Sustainable Agriculture, Khon Kaen University, Khon Kaen 40002, Thailand; 3 Mitr Phol Innovation & Research Center, 399 Moo 1 Khok Sa-At Sub-District, Phu Khiao District, Chaiyaphum 36110, Thailand; jamnank@mitrphol.com (J.K.); suntitr@mitrphol.com (S.R.)

**Keywords:** yield stability, AMMI analysis, GGE biplot, agricultural sustainability, economic returns

## Abstract

Black soybean is a high-value alternative crop that can offset declining yellow soybean prices in Thailand. Currently, “Sukhothai3” is the only commercial variety produced through conventional breeding over the past three decades. Its low first-pod height (FPH) limits mechanized harvesting and reduces production efficiency. Recently developed elite RILs have shown improved FPH, grain yield, and protein content (34.88–41.22%), but their yield stability and adaptability across environments remain unevaluated. This information is essential for establishing an effective bi-directional seed rotation system while maintaining seed quality, quantity, and genetic purity. This study evaluated five black soybean RILs across eleven trial environments in the major production zones of Northeast Thailand, where the tropical savanna climate poses significant constraints on crop production. Field experiments used a randomized complete block design with four replications per location. Yield stability and adaptability were assessed using AMMI1 and GGE biplot analyses. BSCM41 emerged as the most stable and widely adaptable genotype, consistently outperforming the commercial check across environments and seasons. It exhibited adequate FPH for mechanized harvesting (>10 cm), rapid vegetative development, and short growth duration (88–91 days), reducing exposure to late-season heat stress and excessive rainfall. Higher seeds per pod, greater 100-seed weight, and a superior harvest index further support yield formation under marginal conditions. BSCM41 maintained high yields in both dry and rainy seasons (1524.73 and 1633.26 kg/ha, respectively), meeting the requirements for an efficient bi-directional seed rotation system. These findings can inform future breeding strategies and production management in comparable tropical environments.

## 1. Introduction

Black soybean (BS) [*Glycine max* (L.) Merrill] has superior nutritional value and unique bioactive properties, and it is widely used in pharmaceutical products and functional foods in Asia. The seed coat of BS accumulates anthocyanins at concentrations ranging from 280.16 to 2633.45 mg/100 g [1,2]. Medically, anthocyanins have been linked to antioxidant activity, reduced cancer risk, anti-inflammatory and antimicrobial properties, and mitigation of DNA degradation during cellular metabolism [3,4,5]. The phenolic profile of BS plays a critical role in enhancing plant tolerance to abiotic stress [6,7], particularly by delaying seed water imbibition during germination under flooded conditions [8]. Over the past decade, numerous studies have shown that anthocyanins are associated with tolerance to heat, drought, salinity, and waterlogging stress in plants [9,10,11]. Because of these nutritional and functional advantages, BS commands a market price about three to four times that of yellow soybean (YS), positioning it as a valuable ingredient for future premium pharmaceutical and nutraceutical products [12,13]. In addition, black soybean may serve as an alternative cultivar with enhanced tolerance to multiple abiotic stresses, contributing to crop productivity under climate change [14].

Over the past three decades, “Sukhothai3” remained the only commercial BS variety developed through conventional breeding [15]. However, long-established crop cultivars often showed limited adaptability to increasingly variable environmental conditions and modernized production systems [16]. Since 2025, Chinnarat et al. [15] have successfully developed elite BS lines with improved first pod height (FPH), grain yield, and nutritional traits through conventional breeding. Preliminary yield trials (F_7_ generation) showed promising yield potential (1.60–1.86 t/ha), early maturity (86–107 days), and optimal FPH in several recombinant inbred lines (RILs), including SJ5×KKUSB-108-25-2-1, SJ5×KKUSB-108-30-3-7, NSW1×KKUSB-108-49-3-6, and CM60×KKUSB-108-64-4-8. These RILs exhibited intermediate protein content, ranging from 34.88% to 41.22%, in both rainy and dry seasons. However, the expansion of black soybean production was constrained by the risk of varietal admixture with yellow soybean. Such admixture could reduce grain and seed quality, particularly the phenolic compound content, a defining trait of black soybean [17]. As a result, releasing the new black soybean variety needed to be accompanied by seed quality control and measures to prevent seed contamination to ensure sustainable, long-term black soybean production.

The new black soybean lines reported by Chinnarat et al. [15] showed promising performance under experimental conditions. However, the G × E interaction necessitated further evaluation of these RILs to reliably assess yield potential and adaptability across diverse environments and to select cultivars suitable for practical production systems.

At the time of this study, the bi-directional seed rotation system (BDSRS) had been implemented to preserve soybean seed viability under tropical conditions in Northeast Thailand [18]. The core concept of BDSRS was to use a single cultivar with high yield stability to drive the production system, thereby ensuring genetic purity and high-quality seed or grain [18]. In Thailand, farmers typically rotated their seeds between two environments: the rainy season in upland areas and the dry season after the rice harvest in lowland paddy fields [19]. Temperature, photoperiod, soil heterogeneity, precipitation, and crop management practices substantially affected soybean yields in Northeast Thailand [19]. Genotype-by-environment interaction (G × E) remained a major challenge for reliably selecting superior varieties [20]. Therefore, suitable black soybean cultivars for BDSRS need to exhibit strong adaptability to both water-limited and high-humidity conditions [18]. In addition, yield stability was a crucial criterion for selecting newly commercial varieties to design more resilient cropping systems that buffer against seasonal variability [21]. Multi-environment trials (METs) are the most widely accepted approach for identifying stable, high-yielding genotypes [20,22].

Additive main effects and multiplicative interaction (AMMI) and genotype-by-environment (GGE) models, combined with ANOVA, have proven particularly effective for interpreting stability parameters [23,24]. For example, a stability analysis conducted in Nigeria demonstrated the effectiveness of selecting two superior lines from 18 tested genotypes, both of which exhibited high yield potential and broad adaptability across diverse environments [20]. Similarly, elite maize hybrids adapted to waterlogging or normal conditions, as well as hybrids with consistently high performance across both environments, have been reported [25]. Collectively, these findings align with numerous studies over the past decades [18,26,27], underscoring the effectiveness of advanced statistical approaches for genotype evaluation across multiple environments to ensure yield stability and confirm suitability for breeding programs and commercial production.

Therefore, this study aimed to investigate the adaptability of BS RILs for grain yield across multiple locations and years. We used an integrative approach that combined nonparametric models and statistical tools. In addition, we emphasized evaluating morphology and yield-related traits consistent with BDSRS in Northeast Thailand, such as optimal first-pod height and early maturity to escape stress. Widely adapted genotypes could facilitate the development of a duo-environment seed rotation system to target suitable production areas while maintaining the purity and quality of yellow soybean seeds. Moreover, specifically adapted varieties could support the identification of geographical indication (GI) production zones or inform environment-specific management strategies to maximize product quality.

Overall, the high market value of the newly developed black soybean lines was expected to provide farmers with an alternative cultivar that could increase farm income and improve quality of life. Furthermore, this initiative could serve as a model for developing and promoting niche-market crop varieties in other crops.

## 2. Results

### 2.1. Effect of Genotype (G), Environment (E), and G×E on Trait Variation

The combined ANOVA showed that environment, genotype, and G × E had highly significant effects (*p* ≤ 0.01) on agronomic traits, yield-related traits, and grain yield (Table 1 and Table 2). The variance component (VC) derived from the expected mean square revealed the main effect of variation for each trait. In these results, environmental factors predominantly affected the phenotypic expression of plant height (38.52%), pods per plant (69.12%), and 100-seed weight (40.62%) (Table 1 and Table 2). Conversely, days to harvest (84.52%), first pod height (70.29%), nodes per plant (59.36%), branches per plant (63.58%), harvest index (63.85%), and grain yield (38.74%) were clearly under stronger genetic than environmental effects (Table 1 and Table 2). This result highlights the importance of yield stability trials across diverse genotypes and environments to capture the full range of key black soybean traits influenced by G × E interactions.

### 2.2. Growth, Development, and Maturity Rate Parameter

**Figure 1:**
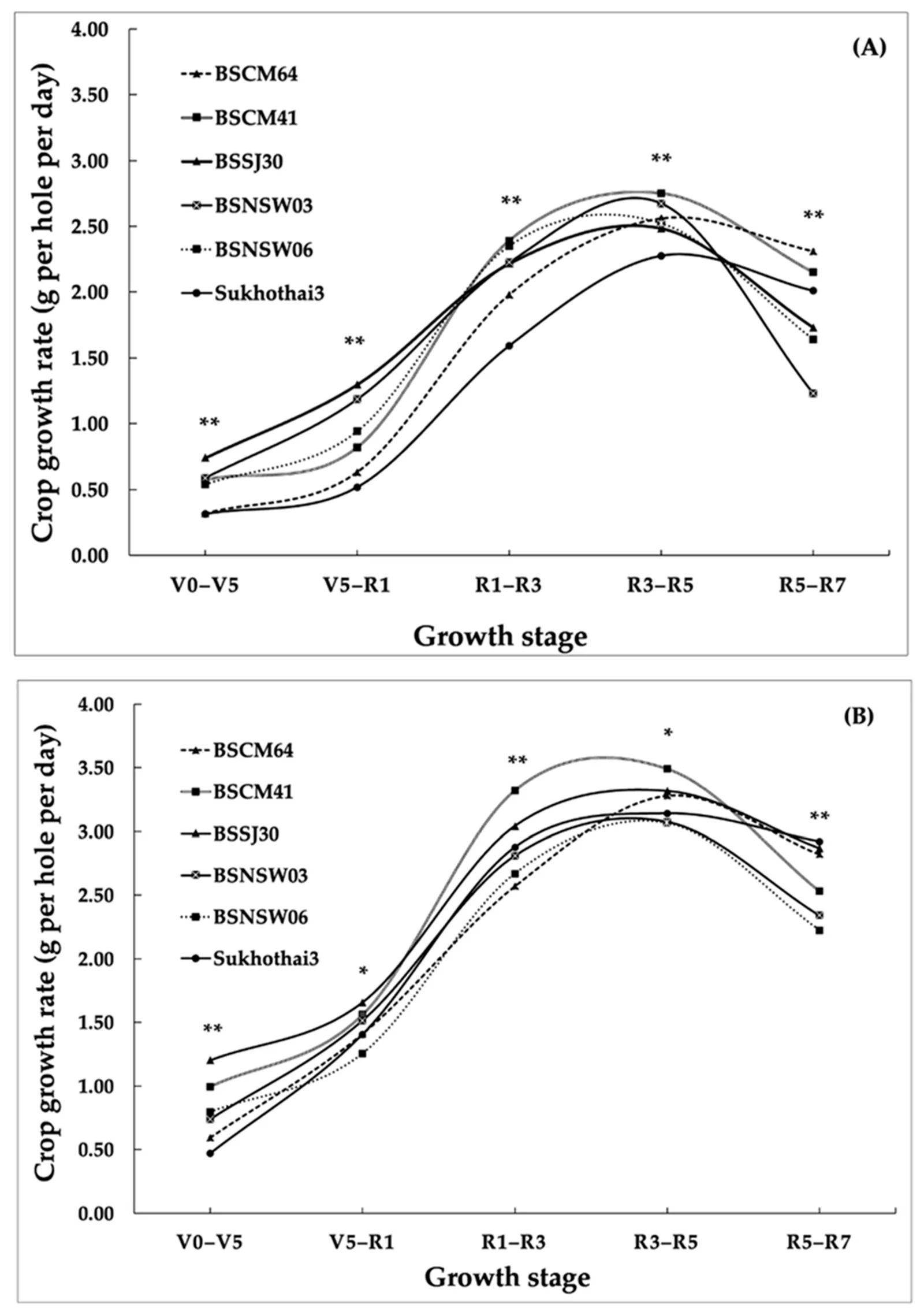
Sigmoid curve pattern based on CGR of six black soybean genotypes across seasons during 2024–2025, combining CGR across six environments in the dry season (**A**) and five in the rainy season (**B**); **, * = significantly different at *p* ≤ 0.01 and 0.05, respectively.

The accelerated growth potential of six genotypes under environmental fluctuations was revealed by the crop growth rate (CGR), which followed a sigmoid curve (Figure 1A,B). Under both wet and dry environmental conditions, all black soybean genotypes showed significantly different CGR trends across growth stages in both seasons (*p* ≤ 0.01 and *p* ≤ 0.05).

In the dry season (Figure 1A), BSSJ30 and BSNSW03 exhibited rapid spatial occupation during the establishment phase (V0–V5). During the transition from the vegetative (V0) to the reproductive phase (R1), all genotypes showed a substantial increase in CGR, with similar trends. During the grain-filling stage, BSCM41, BSNSW03, and BSNSW06 exhibited rapid dry-weight accumulation during the R1–R5 stages, outperforming the other genotypes, whereas BSCM64, BSSJ30, and Sukhothai3 showed a steady, gradual growth pattern (Figure 1A).

In the rainy season (Figure 1B), BSCM41, BSSJ30, and BSNSW03 continued to show strong early growth potential, enabling rapid spatial occupation from V0 to R1, consistent with their dry-season responses (Figure 1A). In contrast, the dry-matter accumulation efficiency of BSCM41, BSNSW03, and BSNSW06 tended to decline as seed filling progressed from R5 to R7, due to accelerated whole-plant senescence (Figure 1B). However, BSCM64, BSSJ30, and Sukhothai3 maintained relatively stable CGR trajectories from the onset of the reproductive phase (R3–R7). This pattern largely mirrored their responses during the dry season (Figure 1A), except for BSSJ30, which exhibited improved adaptability in sustaining CGR under high-moisture conditions. The peak CGR for each line occurred between the R5 and R6 stages, followed by a slight decline toward the harvest stage (Figure 1A,B).

**Figure 2:**
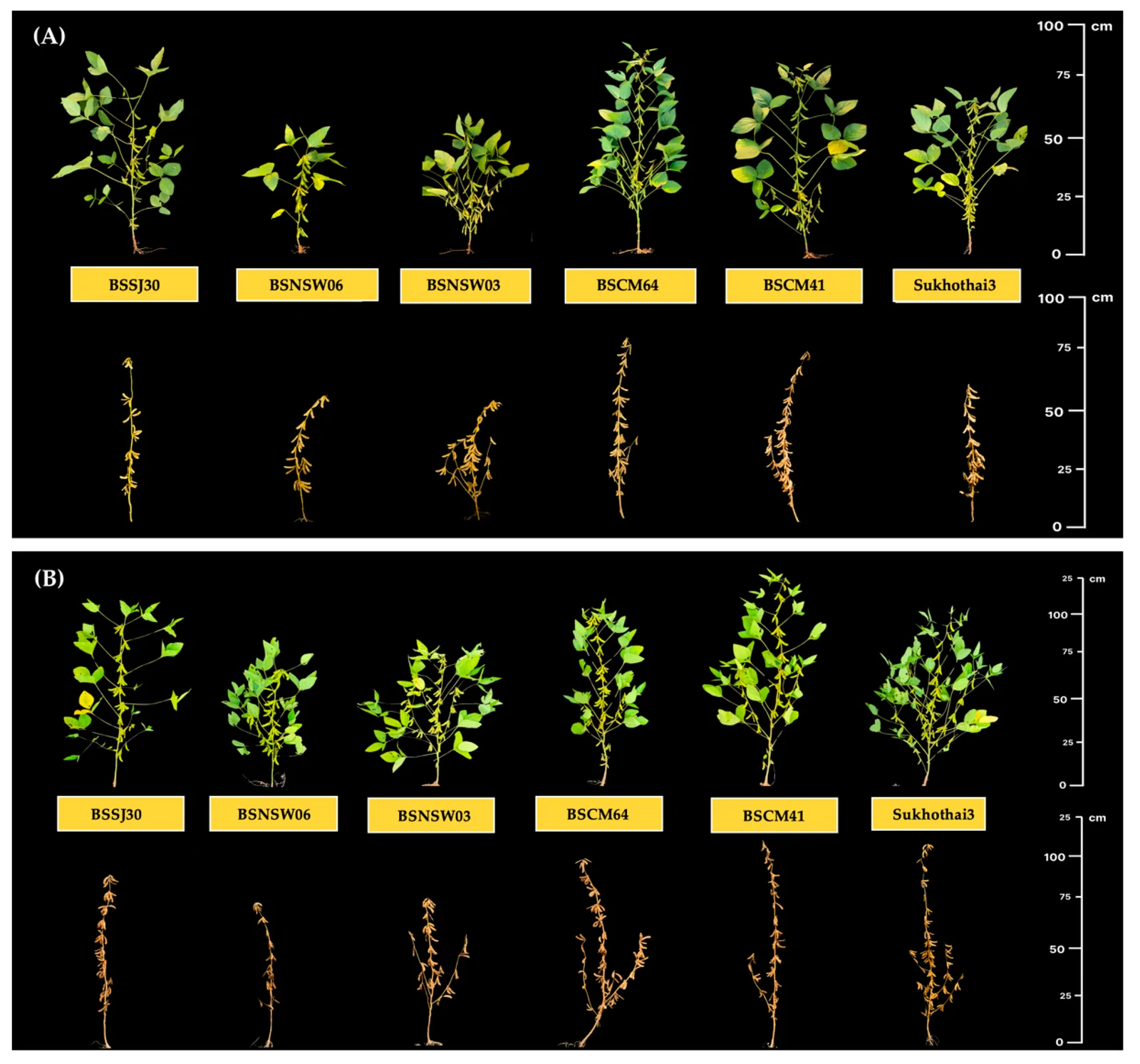
Plant morphology of six black soybean genotypes at the R6 and R8 stages during the dry season (**A**) and the rainy season (**B**) at the Agronomy Field Crop Station, Khon Kaen University, 2024.

Overall, the CGR of all genotypes during the rainy season was markedly higher for dry matter accumulation and biomass production than during the dry season (Figure 2). Conversely, the trend in dry matter accumulation across growth stages was primarily governed by the genotype’s genetic potential. Notably, BSCM41’s growth behavior highlights its potential to rapidly accumulate dry weight over short periods in both the dry and rainy seasons. In contrast, commercial cultivars (Sukhothai3) consistently exhibit slower accumulation, particularly under dry conditions (Figure 2A,B).

### 2.3. Agronomic Traits and Yield Components Variation

The summary table of mean values across eleven environments comprehensively illustrated the yield components of the six soybean genotypes, with all yield-related traits showing highly significant genetic variation at *p* ≤ 0.01 (Table 3).

The harvesting period of the six genotypes ranged from 78.73 to 97.82 days, with BSNSW03 (82.41 days) and BSNSW06 (78.73 days) exhibiting the shortest duration, followed by BSCM41 (90.16 days). The first pod height of five RILs ranged from 9.29 to 13.29 cm, while Sukhothai3 showed the lowest value at 5.28 cm. In addition, the plant height of BSCM64 (71.63 cm) and BSCM41 (73.43 days) was the highest among the genotypes, while BSNSW03 (54.39 cm), BSNSW06 (48.66 cm), and Sukhothai3 (53.27 cm) were the lowest. Despite their relatively shorter plant height compared with the other genotypes, both RILs exhibited a yield-compensatory trait, with higher branching capacities of 3.72 and 3.77, respectively. In contrast, other agronomic attributes of BSNSW03 were markedly inferior to those of Sukhothai3. As a result, the extensive branching observed in Sukhothai3 confers a distinct advantage, increasing the number of pods per plant compared with the other evaluated genotypes. On the other hand, several yield-contributing traits of BSCM41 demonstrated genetic superiority over the commercial cultivar (Sukhothai3), such as the number of nodes per plant (15.04 and 13.55, respectively), 100-seed weight (12.97 and 12.60 g, respectively), and seeds per pod (2.54 and 2.29, respectively). However, when considering the overall contribution of yield components to final seed production, the commercial cultivar (Sukhothai3) exhibited the highest potential among all RILs, as clearly reflected by its superior harvest index (0.31), followed by BSNSW06 (0.29), BSNSW03 (0.27), and BSCM41 (0.27) (Table 3). The results indicated that Sukhothai3 efficiently translocated and allocated assimilates to yield formation, despite having a lower dry matter accumulation rate than the other genotypes (Figure 1).

### 2.4. Grain Yield Adaptation Analysis Across Multiple Environments

#### 2.4.1. Grain Performance Across Multiple Environmental Variations

**Figure 3:**
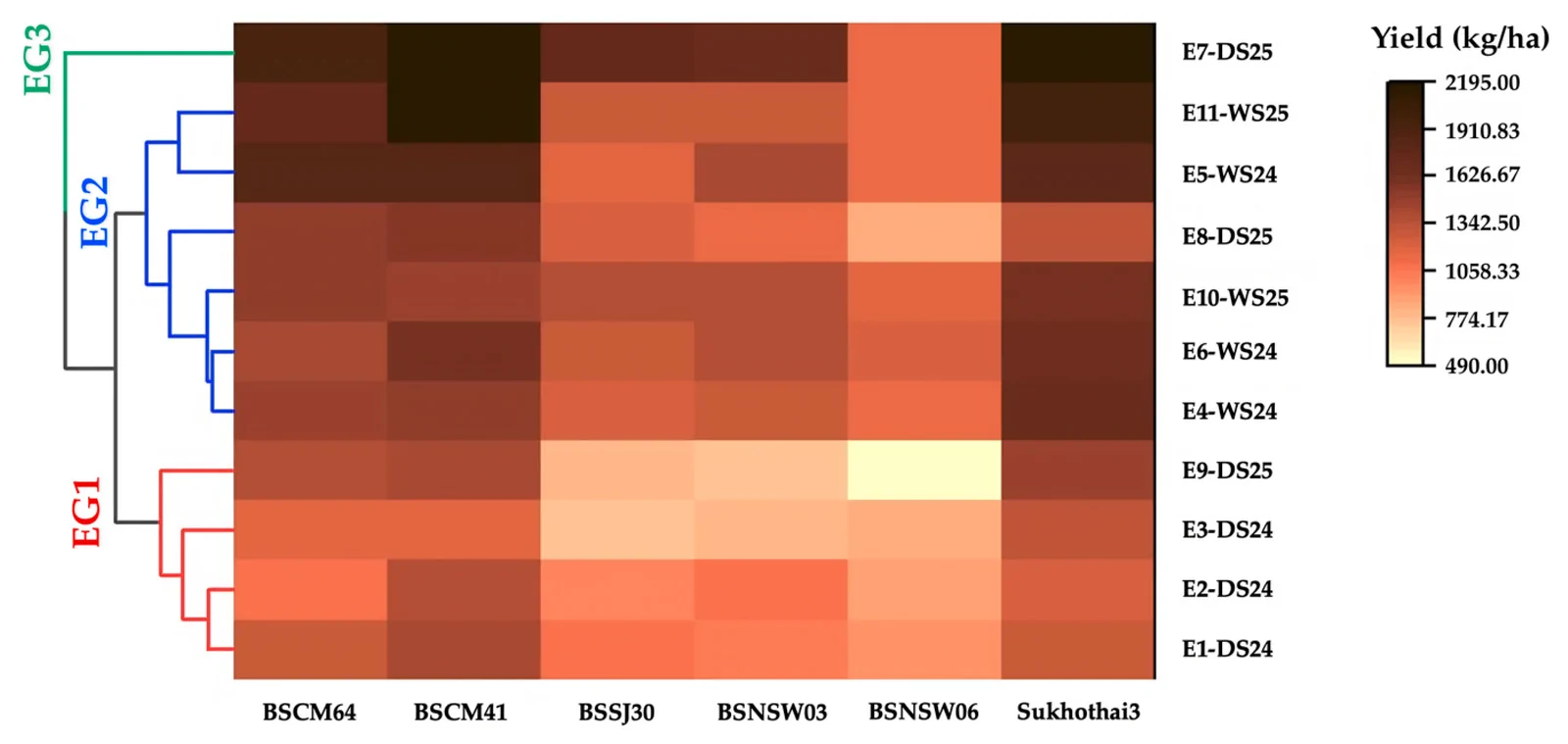
Classification of grain yield performance (kg/ha) for six genotypes across eleven environmental conditions using a heat map dendrogram (Software OriginPro, Version 2025).

The GEI heatmap showed the relationship between each environment and genotype. The eleven environments were grouped into three clusters based on yield performance, including the high-average-grain-yield group (E7-DS25), the low-grain-yield group (E1-DS24, E2-DS24, E3-DS24, and E9-DS25), and the medium-grain-yield group (E4-WS24, E5-WS24, E6-WS24, E8-DS25, E10-WS25, and E11-WS25) (Figure 3). These results show that even a single contrasting seasonal factor was sufficient to generate significant yield variation, providing a valuable basis for identifying soybean genotypes with both superior productivity and stability under G × E in northeast Thailand.

Grain yield performance of six black soybean genotypes varied considerably across environmental groups (EGs). The mean increased markedly from 1126.31 kg/ha in low-yield environments to 1845.96 kg/ha in high-yield environments (Table 4). Among the genotypes, Sukhothai3 consistently produced the highest yields across EG1 and EG2, achieving 1362.39 and 1632.11 kg/ha, respectively. Sukhothai3 had the highest overall mean yield (1592.83 kg/ha), indicating broad adaptability and strong responsiveness to favorable conditions. Similarly, BSCM41 exhibited remarkably high yields and stable performance across environments (1582.89 kg/ha). Notably, BSCM41 maintained high productivity even under unfavorable conditions (1349.00 kg/ha in EG1). It also showed the highest yield under favorable conditions (2190.21 kg/ha in EG3), surpassing all other genotypes in that environment. In contrast, BSNSW06 performed poorly across environments, averaging only 1009.59 kg/ha. Interestingly, BSSJ30 and BSNSW03 showed comparable mean yields (1181.86–1200.21 kg/ha) but differed in adaptive patterns: BSSJ30 responded positively to high-yield environments, whereas BSNSW03 performed better under moderate conditions (Table 4).

Overall, the six genotypes exhibited distinct patterns of broad or specific adaptability, reflecting the influence of genotype × environment interaction (GEI) (Table 4). These results indicated that black soybean yield varied with environmental conditions and that appropriate environmental management can substantially increase productivity. Importantly, BSCM41 emerged as the most promising candidate for cultivation to enhance black soybean productivity, as it performed closely to the commercial genotype (Sukhothai3) even in the low-yield environment group (Table 4), which predominantly represents production conditions in Northeast Thailand.

#### 2.4.2. Grain Yield Performance Across Seasons Parameter

The average grain yield of six black soybean genotypes across dry and rainy environmental conditions in 2024 showed highly significant differences at *p* ≤ 0.01 (Table 5). Average grain yield across environments ranged from the lowest, 1014.46 kg/ha (E1-DS24), to the highest, 1524.80 kg/ha (E6-WS24). BSCM41 and Sukhothai3 exhibited the highest, clearly superior yield potential (1485.84 and 1488.91 kg/ha), whereas BSNSW06 recorded the lowest mean yield (1022.65 kg/ha). In the dry season, BSCM41 and Sukhothai3 consistently achieved superior grain yields under low relative humidity and supplemental irrigation, particularly in environment E3-DS24, where they yielded 1619.35 and 1632.41 kg/ha, respectively. In contrast, during the rainy season, BSCM64 (1852.62 kg/ha) and BSCM41 (1820.47 kg/ha) achieved the highest mean yields at E6-WS24, with no statistically significant difference from the commercial check variety (1771.15 kg/ha). Notably, BSCM64 exhibited specific adaptation to environments with uniformly high soil moisture throughout the growing period (E4-WS24 and E6-WS24), whereas BSCM41 and Sukhothai3 combined high yield potential across both seasonal conditions, reflecting broader adaptability and stability.

In 2025, average grain yield across all environments increased compared with 2024, peaking at 1845.96 kg/ha in the dry season (E7-DS25) and 1606.00 kg/ha in the rainy season (E11-WS25). The mean grain yields for BSCM41, Sukhothai3, and BSCM64 remained the highest at 1679.93, 1696.76, and 1545.03 kg/ha, respectively (Table 6). Under wet conditions, E11-WS25 proved the most favorable environment, supporting the highest yield potential across all six genotypes. Notably, Sukhothai3 and BSCM41 (2158.87 and 2005.30 kg/ha, respectively) were consistently suitable for cultivation under rainy conditions and achieved the highest yields in this environment, followed by BSCM64 (1762.70 kg/ha). By contrast, both BSCM64 and Sukhothai3 maintained yield levels even under continuous heavy rainfall throughout the growing season in E10-WS25 (1520.70 and 1611.30 kg/ha, respectively). However, BSCM41 outperformed under dry-season conditions, particularly in E7-DS25 (2190.21 kg/ha), E8-DS25 (1562.18 kg/ha), and E9-DS25 (1177.88 kg/ha), consistent with the productivity observed in the commercial cultivar Sukhothai3 (Table 6).

The findings clearly showed that genotypes BSCM41, BSCM64, and Sukhothai3 had the highest yield potential under consistent soil moisture throughout the growth cycle, particularly in fallow fields following sugarcane ratoon removal (E6-WS24 and E11-WS25) during the rainy season. In contrast, genotype BSCM41 showed superior adaptive capacity under water-limited conditions with fluctuating temperatures, highlighting its suitability for dry season cultivation (E3-DS24 and E7-DS25). Across both seasons, genotypes BSSJ30 and BSNSW06 exhibited pronounced seasonal variability in yield performance, indicating high sensitivity to environmental fluctuations. Conversely, BSCM41 and Sukhothai3 consistently maintained high productivity across sugarcane-dismantling areas in the rainy season and in lowland paddy fields in the dry season, underscoring their broad adaptability and yield stability. As a result, these genotypes demonstrate reliability for integration into a bi-seasonal seed rotation system.

#### 2.4.3. Yield Stability and Adaptation Parameter

**Figure 4:**
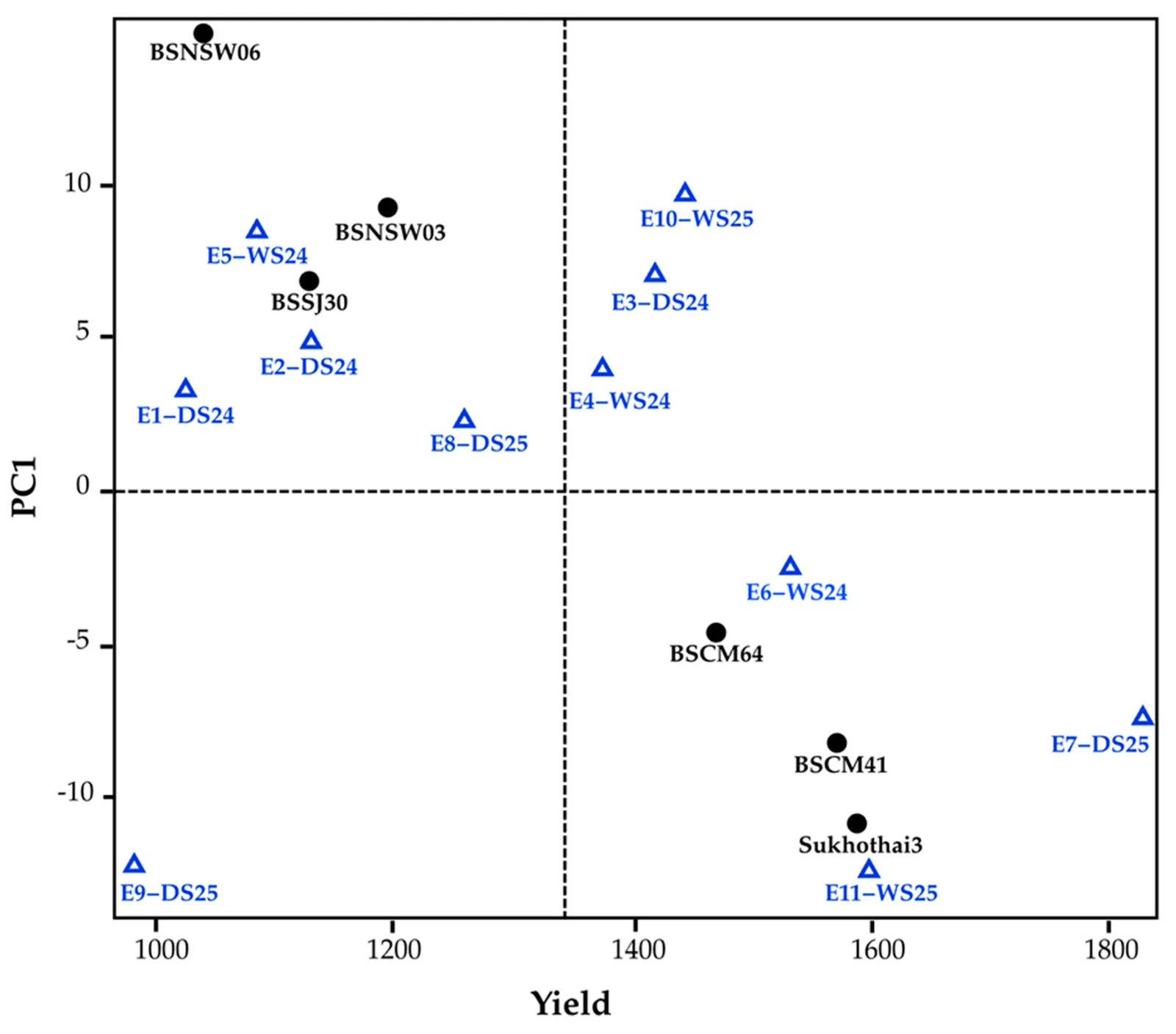
AMMI1 for comparative grain yield average performance of six genotypes across eleven environments: (E1-DS24) Phu Khiao District, Chaiyaphum Province, in dry season 2024; (E2-DS24) Chum Phae District, Khon Kaen Province, in dry season 2024; (E3-DS24) KKU, Khon Kaen Province, in dry season 2024; (E4-WS24) KKU, Khon Kaen Province, in wet season 2024; (E5-WS24) Ban Thaen District, Chaiyaphum Province, in wet season 2024; (E6-WS24) Phu Khiao District, Chaiyaphum Province, in wet season 2024; (E7-DS25) KKU, Khon Kaen Province, in dry season 2025; (E8-DS25) Chum Phae District, Khon Kaen Province, in dry season 2025; (E9-DS25) Ban Thaen District, Chaiyaphum Province, in wet season 2025; (E10-WS25) KKU, Khon Kaen Province, in wet season 2025; (E11-WS25) Chum Phae District, Khon Kaen Province, in wet season 2025.

The AMMI1 biplot revealed a clear positive association between principal component 1 (PCA1) and grain yield potential (Figure 4). BSSJ30, BSNSW03, and BSNSW06 were classified as below-average performers across all environments, as indicated by their placement to the left of the *Y*-axis. In contrast, Sukhothai3 exhibited the highest grain yield among all genotypes, positioned furthest to the right on the *Y*-axis, followed by BSCM41 and BSCM64. Similarly, the environments’ yield potential differed markedly, with E7-DS25 providing the most favorable conditions and yielding the highest mean yield across genotypes, followed by E11-WS25 and E6-WS24. Yield stability is reflected by the proximity of PCA1 values to zero on the *X*-axis. BSSJ30 (from the lowest mean yield group) and BSCM64 (from the highest mean yield group) were less influenced by genotype × environment interaction effects than other genotypes (Figure 4).

**Figure 5:**
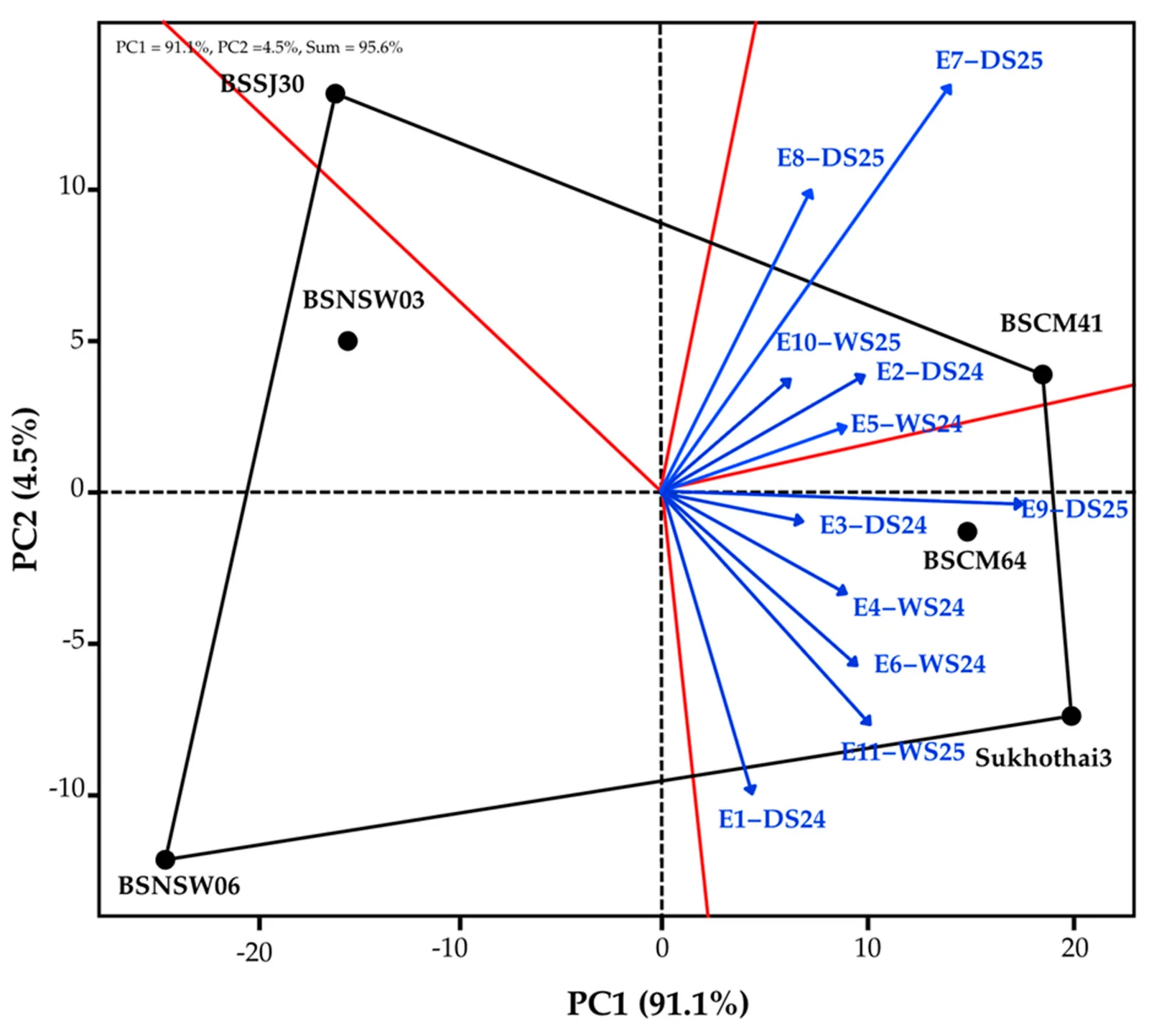
GGE biplot analysis for evaluation of genotype adaptability and yield performance across eleven environments: (E1-DS24) Phu Khiao District, Chaiyaphum Province, in dry season 2024; (E2-DS24) Chum Phae District, Khon Kaen Province, in dry season 2024; (E3-DS24) KKU, Khon Kaen Province, in dry season 2024; (E4-WS24) KKU, Khon Kaen Province, in wet season 2024; (E5-WS24) Ban Thaen District, Chaiyaphum Province, in wet season 2024; (E6-WS24) Phu Khiao District, Chaiyaphum Province, in wet season 2024; (E7-DS25) KKU, Khon Kaen Province, in dry season 2025; (E8-DS25) Chum Phae District, Khon Kaen Province, in dry season 2025; (E9-DS25) Ban Thaen District, Chaiyaphum Province, in wet season 2025; (E10-WS25) KKU, Khon Kaen Province, in wet season 2025; (E11-WS25) Chum Phae District, Khon Kaen Province, in wet season 2025.

The GGE biplot (PCA1 vs. PCA2) in the “Which-won-where” pattern captured most of the variance attributable to genotype, environment, and GEI (Figure 5). Genotype and environment explained 95.60% of the total variance, supporting the selection of stable, high-yielding genotypes across diverse, representative environments in this study. Genotype BSCM64 was positioned near the center of the all-environment biplot, indicating high stability and moderate yield. Similarly, although genotype BSNSW03 performed stably across environments, its yield potential was below the overall mean. In addition, BSCM41 was broadly adaptable across most environments and had peak yield potential close to that of Sukhothai3.

The length of the environmental vector is proportional to the standard deviation (Sd2) of the average yield across genotypes evaluated in that environment. E7-DS25 and E9-DS25 exhibited the longest environmental vectors among the dry-season environments, indicating the greatest yield discrimination among genotypes. In contrast, E11-WS25 was the only rainy-season environment showing high yield variation across all genotypes. E3-DS24 (dry season) and E10-WS25 (wet season) exhibited relatively short environmental vectors, indicating limited ability to discriminate yield variation among the BS RILs. The results emphasized the outstanding performance of BSCM41 and its suitability as a representative genotype for effective cultivation in the bi-directional seed rotation system (Figure 5). This summary closely matched the yield performance patterns presented in Table 5 and Table 6.

## 3. Discussion

### 3.1. Effect of Environment, Genetics, and G × E Variance for Multi-Traits

Genotype ranks across environments change, complicating reliable selection of soybean varieties for commercialization because genotype performance is inherently inconsistent [28].

The highly significant G × E effect observed here underscores the need to select superior genotypes within representative environmental groups that adequately capture the soybean production area in Northeast Thailand [18,19,29], consistent with earlier reports of pronounced genotype-by-environment effects on key yield-related traits [20,30]. In contrast, variation in traits with more complex genetic architectures was governed primarily by genotypic effects rather than by environmental or GEI influences (Table 1 and Table 2). The larger MS for genotype than for G × E was driven by yield differences among the plant materials included: early-maturing genotypes with relatively low yield, such as BSNSW06, and later-maturing genotypes with higher yield, such as Sukhothai3. This consistently large variation in genetic yield potential in all environments resulted in a dominant genotype main effect, with G accounting for 38.74% of the estimated variance compared with 8.69% for G × E. Nevertheless, the highly significant G × E interaction indicates that genotype performance was not completely consistent across environments. Although the overall ranking was relatively stable between environments, ranking changes in G × E effects occurred particularly among genotypes with similar maturity and yield potential (Table 5 and Table 6). Therefore, the relatively small G × E component does not mean environmental evaluation can be completely omitted. Multi-environment trials remain important for identifying genotype-specific adaptation and high-yielding environments, while the GGE biplot (Figure 5) helps minimize the number of environments required for future trials by identifying representative and discriminating environments. This pattern likely reflects the extensive allelic recombination generated by the good × good crossing scheme used in this study, in which the germplasm KKUSB-108, carrying a black seed coat and high first pod height, was crossed with elite Thai commercial cultivars [15]. Combining two agronomically strong but genetically divergent parents in this way would have broadened allelic reshuffling and increased phenotypic divergence among the resulting genotypes [31,32]. Such divergence is consistent with the elevated genotypic variance relative to other variance components observed for several high-heritability quantitative traits in this study [33,34,35,36].

This pronounced genetic divergence is advantageous for breeding because it increases the likelihood of identifying novel allelic and favorable trait combinations, such as introgressing a black seed coat and high first pod height, alongside desirable agronomic backgrounds from elite parents that support yield stability across contrasting environments [37,38,39,40]. These results further support the effectiveness of pedigree selection in early segregating generations for identifying superior recombinant inbred lines that combine these target traits, as demonstrated by Chinnarat et al. [15].

### 3.2. Idiotypes of Black Soybean for Duo-Seasonal Seed Rotation System

#### 3.2.1. Seasonal Limitation and Adaptation in Rainfall Conditions

Soil characteristics, light intensity, and rainfall distribution patterns largely constrained the growth and yield performance of commercial soybean cultivars under high-rainfall conditions [41]. Fertile sandy-loam and clay-loam soil textures appear to provide a favorable environment for soybean production during the rainy season [18,42]. This aligns with the high mean yields observed in E6-WS24 and E11-WS25, which plot above the vertical line on the AMMI1 biplot (Figure 4). Large pore spaces in sandy particles promote rapid drainage and aeration [43], while the loam and clay fractions provide sufficient organic matter to support rhizosphere microbial activity [44]. By contrast, heavy rainfall during the germination stage (June), particularly in E10-WS25 (Figure S2D), caused early seedling stress, reducing plant survival and stunting growth [45]. Yield losses were further exacerbated by seed decay due to fungal infection when harvest coincided with peak rainfall in September [46]. These results are consistent with the relatively low yield performance of BSNSW03 and BSNSW06, despite the overall increase in yield potential across all genotypes in E6-WS24 compared with E4-WS24 in the same year (Table 5). Moreover, if the peak crop growth rate (CGR) occurs during a shortened dry matter accumulation phase under stress, both source capacity and sink activity are severely compromised, resulting in low productivity [47,48]. However, large-scale dry-season production systems remain primarily dependent on seeds produced during the rainy season via a BDSRS [18]. As a result, an effective rainy-season seed production strategy for black soybean must integrate strong genetics with appropriate crop management to overcome environmental constraints.

Hence, production systems established at the onset of the rainy season (June), such as E4-WS24, E6-WS24, and E10-WS24, should use cultivars with rapid early growth potential while maintaining a slow, steady seed-filling phase to tolerate transient stress during heavy rainfall [49]. High-yielding genotypes such as Sukhothai3 and BSCM 64 exemplify this desirable combination (Figure 1B). Their positions in the GGE biplot (Figure 5) align closely with the environmental vectors (E4-WS24 and E6-WS24). In addition, Adie and Krisnawati [50] reported that plant height, branching pattern, number of nodes, pod number, and 100-seed weight are key traits that contribute to soybean adaptability in high-rainfall environments. Moreover, the genotype should exhibit efficient nutrient uptake and carbon partitioning to reproductive sinks under favorable conditions [51]. This finding is supported by the superior performance of yield-related traits and the elevated harvest index recorded in Sukhothai3 (Table 3). However, both limitations, namely low first-pod height and relatively long maturity duration under sufficient moisture conditions [15,52], were also previously reported for the commercial cultivar KKU35 [19]. An extended maturity period may interfere with land preparation for the subsequent sugarcane crop during the late rainy season [18]. Moreover, harvesting from early rainy-season cropping systems (September–October) results in an excessively long storage period before sowing in the subsequent dry season (December) [19]. Prolonged storage of soybean seeds at temperatures above 15 °C may dramatically accelerate seed deterioration and reduce germination capacity, primarily because atmospheric oxygen oxidizes seed lipids [53]. Therefore, achieving BDSRS for high seed quality requires a delayed-planting strategy combined with early-maturing cultivars. This ensures that harvest occurs closer to the actual production window in the dry season (December).

Among the evaluated genotypes, BSCM41 appears particularly promising, combining a short maturity period (<90 days) with the highest CGR during seed formation (R3–R5), outperforming all other genotypes (Figure 1B). It achieves dramatically higher yields than other early-maturing lines, such as BSNSW03 and BSNSW06 (Figure 4). BSCM41 is an attractive alternative because it combines a superior first-pod height of 10 cm (Table 3) with seed yields that consistently exceed the Thailand average [54]. Importantly, its short maturity duration provides growers with greater flexibility to adjust planting dates [55]. This allows the crop to better synchronize with increasingly unpredictable rainfall patterns driven by climate change [56]. However, these attributes must be aligned with an optimal crop window. For instance, E5-WS24 and E11-WS25 were characterized by delayed planting from mid-July to August (Figures S1D and S2E), with V5-R3 stages coinciding with stable rainfall patterns that enhanced early vegetative growth (Figure S1D). The critical reproductive window (R4–R6) occurred under relatively optimal soil moisture following heavy precipitation in October, avoiding both early-season waterlogging stress and late-season rainfall events (Figure S2E). This strategy promotes high physiological seed quality and ensures timely harvesting before it interferes with land preparation for late-rainy-season sugarcane establishment [18].

#### 3.2.2. Seasonal Limitation and Adaptation in Dry Conditions

The dry-season production system is typically located in lowland areas after the rice harvest, when unfavorable conditions prevail and productivity declines. The production area is at high risk of water deficiency, compounded by heavy clay soils that restrict root penetration and lateral root proliferation [57]. Low temperatures and soybean’s sensitivity to shortened photoperiods during emergence and early reproductive stages in December–January (Figure S2A–C) delay germination and canopy establishment, thereby reducing source strength [58,59]. Subsequently, high night temperatures and soil moisture deficits (Figure S1A–C) during the grain-filling stage (March–April) reduce RuBP carboxylase activity and assimilate translocation, resulting in low yields due to seed shriveling [60]. Similarly, extreme heat waves accelerate nocturnal respiration, increasing photosynthate consumed for metabolism and reducing the sugar reserves available for seed filling [61]. Growing degree days (GDD) accelerate the transition between growth stages, shorten the seed-filling period, and contribute to yield decline [62]. Soybean cultivars with extended maturity (>100 days) are more vulnerable to heat stress [19]. Adjusting planting dates in the dry season is challenging because early establishment of growth depends on residual soil moisture from the rainy season [56]. For this reason, breeders should prioritize developing genotypes with robust tolerance to predictable stressors [63]. This aligns with Mandić et al. [64] in Serbia, where similar environmental constraints require soybean cultivars to withstand low temperatures during the vegetative phase and develop deep root systems to cope with water-deficit conditions in the dry season. Genotypes adapted to such environments should also have a short life cycle (<90 days) and a slow CGR during the vegetative phase to minimize water loss, followed by a rapid reproductive growth rate once conditions improve [19,65]. This helps avoid terminal drought stress and ensures high-quality seed for subsequent rainy-season planting [66]. A high branch number is desirable for compensatory yield formation under growth-limiting conditions, maintaining leaf area index (LAI) and enhancing sink capacity [61], as observed in BSNSW03 (Figure 2). BSCM41 similarly maintained superior yield compensation, with more seeds per pod and higher 100-seed weight than other genotypes (Table 3). Wang et al. [67] supported this, noting that these traits confer a superior capacity for yield compensation compared with dependence on the number of pods per plant. This trait is highly plastic and environmentally labile. These findings demonstrate the adaptive efficiency of both genotypes under harsh dry-season conditions, as illustrated by the GGE biplot (Figure 5). Most soybean production areas in Thailand are located in the northeast, under these environmental conditions [19].

### 3.3. Grain Yield Performance and Stability

Genotype-by-environment (G × E) interaction and yield stability analyses remain important for assessing varietal stability and suitability for cultivation across seasons and ecological zones [28]. Although each RIL exhibits distinct strengths in specific environments, BSCM41 and Sukhothai3 are positioned at the vertices of their respective mega-environments [68]. Yield stability across seasons remains the primary criterion for selecting lines for seed production rotation [18]. Four genotypes, including BSNSW03, BSCM41, BSCM64, and Sukhothai3, exhibited exceptionally low environmental sensitivity, as evidenced by their placement near the PCA1 axis in the GGE biplot (Figure 5), consistent with the AMMI1 results (Figure 4). Genetic adaptability is a key determinant of yield consistency across contrasting dry- and rainy-season environments. The “Which-won-where” GGE biplot explained 95.60% of the total variation (G + GE), indicating high interpretive power for identifying ideal genotypes with strong stability [69]. This proportion aligns with previous reports, including 93.60% [20], 89.10% [18], and 86.60% [70].

Regarding yield potential, BSCM41, despite its short maturity, outperformed the commercial cultivars by 2–3% in E8-DS25 (dry season) and maintained competitive yields across most environments (Table 5 and Table 6). Although BSCM64 showed high stability, its yield was usually lower than that of the top-performing genotypes in some locations. BSNSW03 demonstrated the highest stability and a very short maturity (<85 days), yet its low yield remained a constraint (Table 3). Thus, it may be more suitable for inclusion in breeding pipelines aimed at yield improvement or in production systems that use higher plant densities to compensate for low per-plant productivity [65,71]. Furthermore, the GGE biplot (Figure 5) clearly confirmed the poor adaptability of BSSJ30 and BSNSW06. This result went beyond what environmental grouping alone suggested (Table 4), as both genotypes showed low yields and lacked any environmental vector within each sector [20,72].

Furthermore, most environments in the GGE biplot, particularly during the wet season, had relatively short environmental vectors (Figure 5). These findings suggest that future multi-environment trials could be streamlined by eliminating redundant yield-trial environments and focusing on those with high discriminative ability or the longest vectors [68,73], including E7-DS25 and E11-WS25 (Figure 5). Such an approach would improve resource-use efficiency while maintaining effective selection of superior genotypes. In contrast, environments with short vectors but high mean grain yield across genotypes, such as E3-DS24 (dry season) and E10-WS25 (wet season), may be better suited for optimizing crop management practices than for genotype discrimination [74].

Overall, assessing productivity and stability requires multiple complementary analytical approaches to identify ideal genotypes accurately and reliably [24]. This study demonstrated that BSCM41, which combines high yield potential with strong adaptability across dry- and rainy-season production systems, is superior. Consequently, BSCM41 is the most suitable candidate for release as a new black soybean cultivar tailored for seed rotation systems in Northeast Thailand.

### 3.4. Opportunity and Future Perspectives for Research

This study focused on selecting superior, high-yielding black soybeans based solely on grain yield, with particular emphasis on the genetic traits that underpin their adaptation to contrasting tropical environments. The tropical savanna climate in the Northeast region is characterized by high rainfall, intermittent water stress, and pronounced temperature variability [19].

In Europe, growing concerns have been raised about the negative impacts of long-duration cultivars (140–150 days) that face prolonged, continuous pest and disease pressure amid rising global temperatures [75]. In South Asia, excessive rainfall and prolonged drought have been identified as major drivers reshaping food production systems, often resulting in unfavorable growing conditions [76].

**Figure 6:**
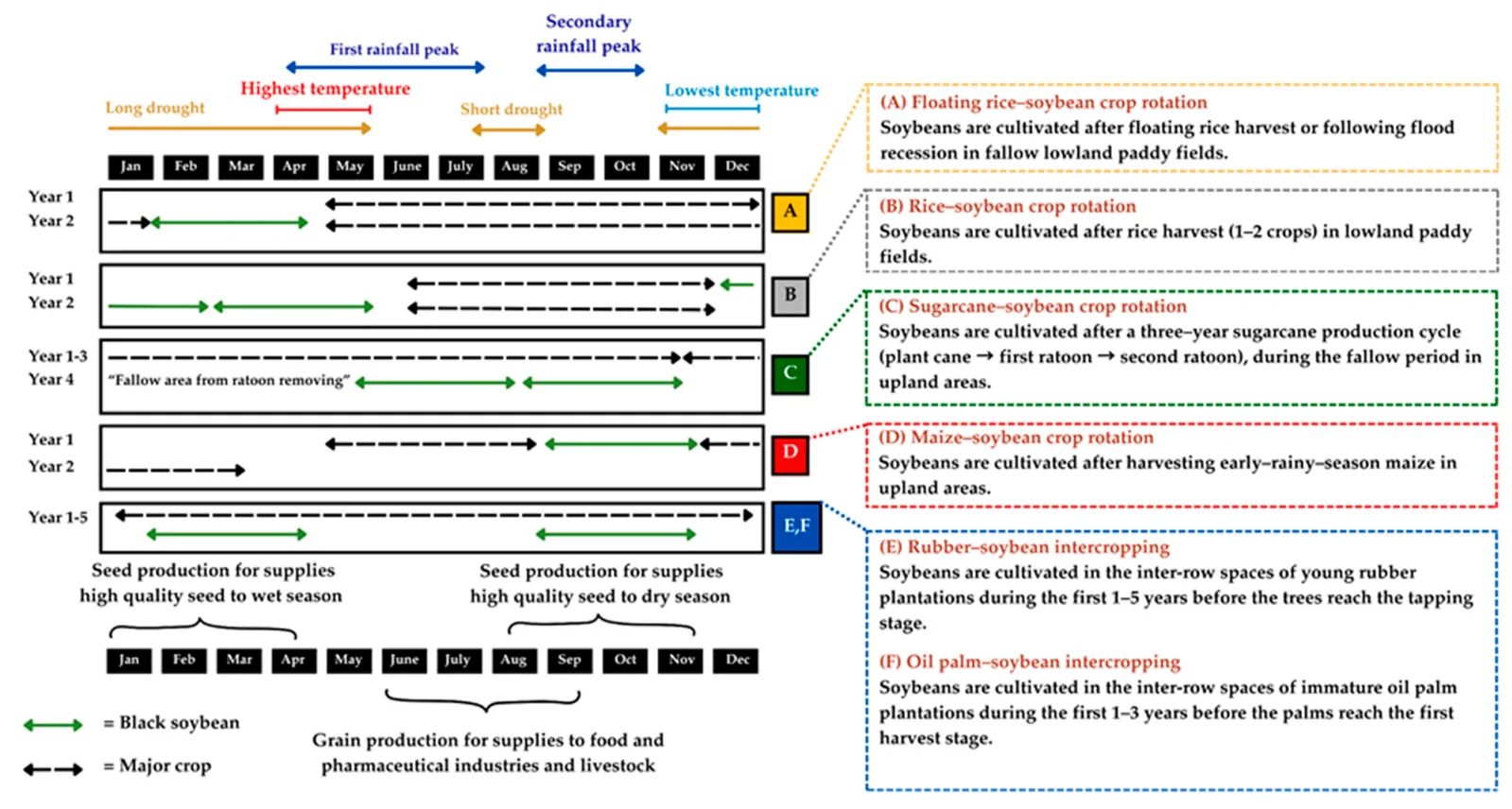
Integrated cropping system guidelines for major economic crops in Thailand and black soybean (cv. BSCM41) to facilitate high-quality seed of soybean circulation under bi-directional seed rotation; (**A**) floating rice; (**B**) lowland rice; (**C**) sugarcane; (**D**) maize; (**E**) rubber tree; (**F**) oil palm; black vectors represent major crops; green vectors represent black soybean.

Consequently, adopting high-yielding, early-maturing soybean cultivars has attracted increasing attention as a risk-mitigation strategy under highly variable environmental conditions [77]. In this study, BSCM41’s short maturity demonstrated its suitability for integration into major cropping systems in Thailand, despite limited planting windows (Figure 6). Floating rice systems in flood-prone areas typically require a prolonged growing period of 7–9 months (May–January) and often yield only about 2 t/ha [78]. Under such conditions, delayed water recession can significantly constrain land preparation and limit the feasibility of cultivating commercial soybean cultivars with maturity durations exceeding 100 days during the dry season. Therefore, BSCM41 may be a suitable early-maturing alternative, enabling soybean production within these narrow time windows and supporting more continuous farm income (Figure 6A,B). In addition, soybean cultivars with maturity durations of 100–140 days require approximately 450–700 mm of cumulative water throughout the growing season [79], whereas early-maturing cultivars (<100 days) evaluated in this study were estimated to reduce water requirements by up to 34–47%. This approach provides an effective strategy for optimizing limited water resources and reducing long-term irrigation costs during the dry season. Previous studies have shown that soybeans can be efficiently cultivated under BDSRS systems by using fallow periods after rice and sugarcane harvests (Figure 6C) [18].

Legume-based crop rotation further improves soil structure and fertility by increasing soil organic matter and nutrient diversity [80]. In the U.S. Midwest, soybeans can fix up to 75 kg of N/ha, reducing reliance on synthetic fertilizers, a major source of N_2_O emissions [81]. The soybean–maize rotation system (Figure 6D) has been shown to enhance soil carbon sequestration by up to 4 Mg CO_2_-eq/ha/yr, offsetting approximately 2.1 Mg CO_2_-eq/ha/yr in emissions [82]. This aligns with net-zero emission strategies aimed at mitigating climate change over the long term [83].

Intercropping black soybean with young rubber or oil palm improves land-use efficiency during the trees’ non-bearing phase, when wide interrow spacing leaves substantial land underutilized during the long juvenile period. Replanted areas remain open and receive full sunlight before the canopy closes [84]. Rather than remaining fallow, this space can be planted with soybeans, generating income before the tree crop begins to fruit. Soybeans are among the most frequently reported intercrops in immature rubber plantations, alongside maize and cassava [85]. Reported outcomes indicate that intercropping soybeans with young oil palm does not inhibit palm growth, and intercropped palms perform comparably to, or better than, sole-planted palms [86]. Together, these findings support intercropping as a practical strategy to maximize land productivity and generate supplementary income during the extended immature phase of rubber and oil palm establishment (Figure 6E,F).

Black soybeans command a market value 3–4 times higher than yellow soybeans, offering substantial economic incentives for farmers [15]. The high anthocyanin content of BSCM41 also offers opportunities to develop a range of value-added products, serving as a key driver for expanding soybean cultivation areas and contributing to long-term food security in Thailand.

However, the effects of environmental fluctuations on the phenolic composition of black soybean remain poorly understood. For this reason, future research should focus on identifying optimal dry- and wet-season environments that promote the accumulation of bioactive compounds in black soybean. This knowledge will enable growers to design more targeted production-management strategies to maximize the genetic potential of BSCM41 for phenolic accumulation and overall productivity. Moreover, integrating these two approaches may also provide a useful framework for breeding and production of black soybeans in other tropical environments with comparable constraints.

## 4. Materials and Methods

### 4.1. Plant Materials

The genotype material consisted of 5 RILs derived from crosses between Thai commercial varieties (YS): SJ5, CM60, NSW1, and KKU35, and KKUSB108 (BS), as well as one commercial variety (Sukhothai3). These RILs were selected in the F_7_ generation using a pedigree selection method based on dark seed coat, high yield, and optimal FPH [15]. Five representative lines used in this study (Table 7) were selected from eight candidate genotypes reported by Chinnarat et al. [15] based on key agronomic criteria: anthocyanin < 100 mg/100 g, maturity duration < 100 days, and plant height < 90 cm (non-lodging). These criteria ensured suitability within the cropping window between major crops in Northeastern Thailand. The traditional commercial BS variety (Sukhothai3) was also high-yielding and highly adaptable to the wild, and it was used as a check in multiple-environment trials (Table 7).

### 4.2. Multi-Location Trials

**Figure 7:**
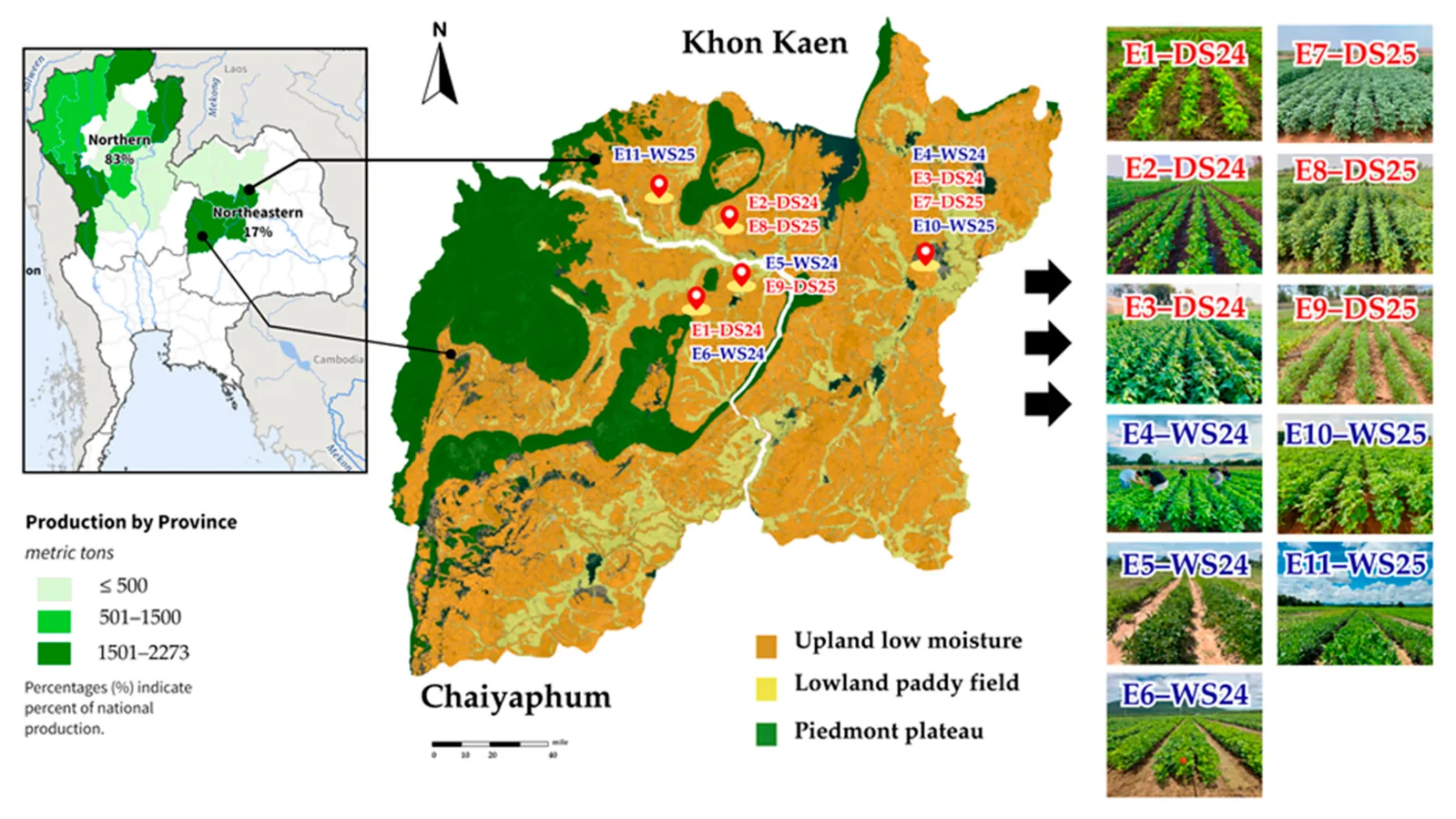
A geographical map (edited using Agri-Map software: https://agri-map-online.moac.go.th) and soybean production information from https://www.fas.usda.gov/data/production/th, accessed on 19 November 2025, demonstrate eleven environmental yield trials: farmer fields in Khon Kaen and Chaiyaphum provinces and the Agronomy Field Crop Station, Khon Kaen. These eleven environments represent a bi-directional seed rotation system between fallow land used for sugarcane (May–October) and rice (December–March) cropping systems in the Northeast region of Thailand. The environments are designated as follows: DS24 = dry season 2024; DS25 = dry season 2025; WS24 = wet season 2024; and WS25 = wet season 2025.

This study was conducted from 2024 to 2025 across eleven locations that differed in environmental factors, including soil chemistry, soil type, soil texture, temperature, precipitation, crop management practices, and planting date (Table 8). The experimental sites were located at farmers’ fields in Khon Kaen and Chaiyaphum provinces and at the Agronomy Field Crop Station, Khon Kaen University, representing major soybean production zones in Thailand and encompassing a wide range of seed rotation systems in the northeast region (Figure 7).

### 4.3. Experimental Design and Crop Management

The experimental design followed a randomized block design (RCBD) with four replications. In the dry season, plot size per experimental unit in the lowland paddy field and the Agronomy Field Crop Station was 54 m^2^ (6 × 9 m), with hole/row spacing of 25 × 50 cm. Plants were thinned to three per hole (240,000 plants/ha). Experimental plots in upland fields varied in size to match the existing sugarcane bed configuration (bed width: 1.5 m). Each of the six sites consisted of two beds, each approximately 54 m^2^ (3 × 18 m), and had the same plant spacing and population density as in a lowland paddy field. Cultivation was conducted during two distinct seasons, representative of seed rotation in the Northeast region: the rainy season (June–September) for post-sugarcane upland environments and the dry season (December–March) for post-rice lowland environments [18]. Immediately after planting, we applied a pre-emergence herbicide, and during the growth period, we applied a post-emergence herbicide and hand-weeded. Fertilizers were initially applied at 14.06 kg/ha (N2-P2O5-K2O) at 21 days after planting (DAPS), with a second application at 50 DAPS of 18.75 kg N2/ha, 37.50 kg P2O5/ha, and 18.75 kg K2O/ha. Insecticides were used as recommended by Thailand’s Department of Agriculture (DOA). Irrigation practices followed DOA recommendations and farmer practices to reflect actual production systems. During the dry season, fields were flood-irrigated five times at 11- to 14-day intervals after planting, with total water input ranging from approximately 300 to 400 mm depending on cumulative evapotranspiration and soil physical properties. During the wet season, rainfall served as the primary water source, supplemented by irrigation during dry spells or when soybean plants exhibited visible signs of water stress.

### 4.4. Data Collection

#### 4.4.1. Growth, Development, and Maturity Rate Parameters

The relationships among first pod height (FPH), days to harvest (DTH), plant height (PH), and crop growth rate (CGR) were examined to assess growth potential and maturity rate across five soybean developmental stages (e.g., V0–V5, V5–R1, R1–R3, R3–R5, and R5–R7). DTH was recorded as the number of days after planting to physiological maturity (R8). FPH and PH were measured from the early part of the main stem above ground to the first node attached to a pod and to the highest tip of the young leaf, respectively. CGR was calculated based on the average dry matter of three randomly selected holes per plot using Equation (1). The allocation of photosynthate to grain yield was assessed by measuring the harvest index (HI) using the same collection method as CGR, but sampling only at the R7 stage and applying Equation (2).

(1) $$CGR\;(g\;per\;hole\;per\;day)\;=\;\frac{TDM2-TDM1}{T2-T1}$$

where TDM1 and TDM2 are the total dry matter of soybean plants at V5, R1, R3, R5, and R7, respectively. T1 is the time of the first sampling, and T2 is the time of the second sampling [87].

(2) $$HI=\frac{Y}{TDMA}$$

where TDMA is total dry matter accumulation (g/hole), and Y is total grain yield (g/hole). This formulation was adapted from the HI of rice [88].

#### 4.4.2. Yield-Related Trait Parameters

Yield-related traits were measured on ten randomly selected plants per plot, and the measurements were averaged to provide representative data. Number of nodes per plant (NP), branches per plant (BP), pods per plant (PP), seeds per pod (SP), and 100-seed weight (100SW) were used to describe yield performance for each genotype. Grain yield (GY) was determined by sampling three square meters of plants from each plot. The plot GY was then converted to kg/ha. The seeds were gradually reduced to a moisture content of 14% by sun-drying before yield potential was calculated [89].

### 4.5. Data Analysis

#### 4.5.1. Combined Analysis of Variance

The initial process required a homogeneity test for each parameter, including agronomic traits, grain yield, and yield-related traits. After that, a combined analysis of variance (ANOVA) for an RCBD across eleven environments was conducted using the Statistix 10© program (version 10.0; 1985–2013) (Analytical Software, Tallahassee, FL, USA). The means for each trait were compared using Fisher’s least significant difference (LSD) in R-Stat [90].

#### 4.5.2. Expected Mean Square

The Expected Mean Squares (EMSs) were derived using the method described by McIntosh [91] to estimate the variance components (σ^2^E, kG, and σ^2^GE) in the mixed-model term (Table 9). These components were then used to estimate the main effect on the expansion of trait variation, with genotypes treated as fixed effects and environments as random effects.

#### 4.5.3. Stability Analysis

Yield stability was assessed using non-parametric analyses to build a model diagram that visually demonstrates G × E interactions via principal component patterns (AMMI1 and GGE biplot models), implemented in R software version 4.4.2 [90]. AMMI analysis will facilitate a comprehensive understanding of the GEI structure [30]. The GGE biplot was established using the association between the first and second principal components (PC1 and PC2) derived from environment-centered grain yield data [92]. GGE and AMMI results clarify the nature of GEI, providing key criteria for identifying both broadly adaptable varieties and those adapted to specific environments.

## 5. Conclusions

This study demonstrates that BSCM41 combines high yield stability with broad adaptability, highlighting its potential as a promising candidate genotype for trial integration into bi-directional seed rotation systems across contrasting cropping seasons in Northeast Thailand. GGE biplot analysis showed that Sukhothai3 (check) and BSCM64 were best adapted to optimal-temperature, high-moisture environments, whereas BSCM41 performed closely with the commercial cultivars under drought and fluctuating temperature conditions and clearly outperformed the other RILs in both dry and rainy seasons. AMMI1 confirmed BSCM41’s superior stability alongside Sukhothai3, reinforcing its role as a rotational partner. Its optimal first pod height (>10 cm), rapid biomass accumulation, and short harvest window (90 days) give BSCM41 strong drought and excess-rainfall-escape potential, filling planting windows where Sukhothai3 is less reliable. This makes BSCM41 a practical option for expanding black soybean production and strengthening food security.

## Figures and Tables

**Table 1:** Mean squares from a combined analysis of variance for plant height (PH), days to harvest (DTH), first pod height (FPH), nodes per plant (NP), and branches per plant (BP) of six soybean genotypes across eleven environments, showing genotype (G), environment (E), and genotype-by-environment (G × E) interaction.

Source	df	PH		DTH		FPH		NP		BP	
		MS	VC (%)	MS	VC (%)	MS	VC (%)	MS	VC (%)	MS	VC (%)
E	10	2767.16 **	38.52	167.78 **	8.46	34.08 **	13.61	20.25 **	13.13	4.24 **	9.42
Reps./E	33	62.97		1.33		1.19		0.34		0.29	
G	5	4823.97 **	36.36	3069.76 **	84.52	315.04 **	70.29	169.39 **	59.36	49.92 **	63.58
G × E	50	144.93 **	8.47	19.76 **	5.71	3.57 **	6.60	4.39 **	13.42	1.04 *	10.87
Error	165	45.87		1.03		0.91		1.00		0.28	
CV (G)		12.96		1.28		11.24		8.86		20.96	
CV (E)		19.66		4.91		19.34		16.30		39.77	
CV (GE)		11.06		1.12		9.80		7.77		20.70	

CV = coefficient of variation (%); *, ** = significantly different at *p* ≤ 0.05 and 0.01, respectively; Reps./E = replication across environments; df = degrees of freedom; MS = mean square; VC = variance component percentage.

**Table 2:** Mean squares from the combined analysis of variance for pods per plant (PP), 100-seed weight (100SW), yield, harvest index (HI), and seeds per pod (SP) across eleven environments for six soybean genotypes, showing genotype (G), environment (E), and genotype × environment (G × E) interaction.

Source	df	PP		100SW		Grain yield		HI		SP	
		MS	VC (%)	MS	VC (%)	MS	VC (%)	MS	VC (%)	MS	VC (%)
E	10	2723.10 **	69.12	19.23 **	40.62	383,488.90 **	36.35	4.34 × 10^−3^ **	13.51	0.18 **	5.06
Reps./E	33	72.80		0.42		1594.40		1.89 × 10^−4^ **		0.03	
G	5	25,526.30 **	17.49	26.54 **	29.66	74,071.70 **	38.74	3.67 × 10^−2^ **	63.85	1.95 **	34.22
G × E	50	9373.80 **	5.55	1.36 **	13.99	1974.00 **	8.69	7.30 × 10^−4^ **	11.72	0.09 **	12.14
Error	165	7523.50		0.28		504.20		1.30 × 10^−4^		0.03	
CV (G)		19.59		5.47		18.61		5.12		7.72	
CV (E)		31.44		9.87		20.70		10.08		13.56	
CV (GE)		15.51		4.51		10.46		4.17		7.63	

CV = coefficient of variation (%); ** = significantly different at *p* ≤ 0.01; Reps./E = replication across environments; df = degrees of freedom; MS = mean square; VC = variance component percentage.

**Table 3:** Mean performance of plant height (PH), days to harvest (DTH), first pod height (FPH), nodes per plant (NP), branches per plant (BP), pods per plant (PP), 100-seed weight (100SW), seeds per pod (SP), and harvest index (HI) of six soybean genotypes across eleven environments.

Genotype	Combined Value of Morphological and Yield-Related Traits								
	PH (cm)	FPH (cm)	DTH (Days)	NP	PP	BP	SP	100SW (g)	HI
BSCM64	71.63 AB	9.52 C	97.82 A	14.91 A	53.85 AB	2.77 B	2.19 B	11.67 B	0.25 D
BSCM41	73.43 A	11.47 B	90.16 B	15.04 A	46.12 BC	1.97 C	2.54 A	12.97 A	0.27 C
BSSJ30	66.03 B	13.29 A	96.66 A	11.66 C	36.45 D	2.07 C	2.21 B	11.06 B	0.23 E
BSNSW03	54.39 C	9.29 C	82.41 C	11.78 C	41.73 CD	3.72 A	1.96 C	11.20 B	0.27 C
BSNSW06	48.66 C	9.45 C	78.73 D	10.17 D	27.32 E	1.07 D	2.00 C	11.48 B	0.29 B
Sukhothai3	53.27 C	5.28 D	97.80 A	13.55 B	55.81 A	3.77 A	2.29 B	12.60 A	0.31 A
Mean	61.24	9.72	90.60	12.85	43.55	2.56	2.20	11.83	0.27
F-test	**	**	**	**	**	**	**	**	**
CV (GE)	11.06	9.80	1.12	7.77	15.51	20.70	7.63	4.51	4.17

CV = coefficient of variation (%); ** = significantly different at *p* ≤ 0.01; the same letters within a column indicate not significant by LSD comparison (*p* ≤ 0.01).

**Table 4:** Average grain yield performance of six soybean genotypes within each environmental group (EG).

Genotypes	The Grain Yield (kg/ha)			Mean
	EG1 (Low Yield)	EG2 (Moderate Yield)	EG3 (High Yield)	
BSCM64	1218.95	1536.68	1920.18	1558.60
BSCM41	1349.00	1621.42	2190.21	1582.89
BSSJ30	968.49	1210.43	1756.02	1181.86
BSNSW03	989.44	1251.83	1691.82	1200.21
BSNSW06	869.59	1050.11	1339.52	1009.59
Sukhothai3	1362.39	1632.11	2174.99	1592.83
Mean	1126.31	1383.76	1845.96	

EG1 = mean yield across the E1-DS24, E2-DS24, E3-DS24, and E9-DS25 environments; EG2 = mean yield across the E4-WS24, E5-WS24, E6-WS24, E8-DS25, E10-WS25, and E11-WS25 environments; EG3 = mean yield for the E7-DS25 environment.

**Table 5:** Grain yield performance of six soybean genotypes across six environments during the rainy and dry seasons in 2024, demonstrating the G × E interaction derived from multi-environment effects in northeast Thailand.

Genotype	The Grain Yield (kg/ha) in 2024												
	Dry Season						Rainy Season						Mean
	E1-DS24		E2-DS24		E3-DS24		E4-WS24		E5-WS24		E6-WS24		
BSCM64	1166.22	AB	1250.19	AB	1434.77	B	1493.50	AB	1093.66	BC	1852.62	A	1381.83
BSCM41	1195.67	AB	1403.10	A	1619.35	A	1524.53	AB	1351.92	A	1820.47	A	1485.84
BSSJ30	744.63	C	1072.26	BC	1262.10	BC	1239.97	BC	968.39	C	1156.77	C	1074.02
BSNSW03	814.61	AB	1012.52	BC	1381.19	BC	1279.03	BC	1059.14	BC	1404.40	B	1158.48
BSNSW06	856.78	B	918.47	C	1212.64	C	1129.33	C	875.27	C	1143.38	C	1022.65
Sukhothai3	1308.86	A	1281.76	AB	1632.41	A	1695.50	A	1243.78	AB	1771.15	A	1488.91
Mean	1014.46		1156.38		1423.74		1393.64		1098.69		1524.80		
F-test	**		**		**		**		**		**		
CV (%)	18.52		13.33		7.32		10.01		10.58		10.55		

CV = coefficient of variation; ** = significantly different at *p* ≤ 0.01; the same letters within a column indicate not significant by LSD comparison (*p* ≤ 0.01).

**Table 6:** Grain yield performance of six soybean genotypes across five environments during rainy and dry seasons in 2025, demonstrating the G × E interaction derived from the effect of multi-environment effects in northeast Thailand.

Genotype	The Grain Yield (kg/ha) in 2025										
	Dry Season						Rainy Season				Mean
	E7-DS25		E8-DS25		E9-DS25		E10-WS25		E11-WS25		
BSCM64	1920.18	AB	1496.92	AB	1024.63	B	1520.70	AB	1762.70	B	1545.03
BSCM41	2190.21	A	1562.18	A	1177.88	A	1464.10	B	2005.30	AB	1679.93
BSSJ30	1756.02	B	1229.22	C	794.98	C	1387.40	B	1280.84	C	1289.69
BSNSW03	1691.82	B	1118.07	C	749.43	C	1358.70	B	1291.63	C	1241.93
BSNSW06	1339.52	C	857.89	D	490.46	C	1158.00	C	1136.80	C	996.53
Sukhothai3	2174.99	A	1312.08	BC	1226.54	A	1611.30	A	2158.87	A	1696.76
Mean	1845.96		1262.73		985.97		1416.70		1606.00		
F-test	**		**		**		**		**		
CV (%)	8.50		8.41		12.01		5.78		7.38		

CV = coefficient of variation; ** = significantly different at *p* ≤ 0.01; the same letters within a column indicate not significant by LSD comparison (*p* ≤ 0.01).

**Table 7:** Details of the five recombinant inbred lines and a commercial black soybean variety used in this study.

Genotype	Variety Type	Pedigree	Maturity Type	Total Anthocyanin (mg/100 g)
BSCM64	RILs	CM60×KKUSB-108-64-4-8	Intermediate	105.22
BSCM41	RILs	CM60×KKUSB-108-41-1-7	Early	159.61
BSSJ30	RILs	SJ5×KKUSB-108-30-3-7	Intermediate	125.72
BSNSW03	RILs	NSW1×KKUSB-108-49-3-3	Early	125.51
BSNSW06	RILs	NSW1×KKUSB-108-49-3-6	Early	135.92
Sukhothai3	Check Variety	Sukhothai3	Intermediate	137.98

**Table 8:** The table describes the eleven environments used for evaluation across Northeast Thailand.

Environment *	Season	Soil Type	Total PAR (MJ/m^2^/Day)	Total Rainfall (mm)	T-Min. (°C)	T-Max (°C)
E1-DS24	Dry 2024	Loam	8.44	71.43	19.47	32.32
E2-DS24	Dry 2024	Clay	8.28	69.29	21.10	34.45
E3-DS24	Dry 2024	Loamy Sand	8.39	58.42	21.08	34.46
E4-WS24	Rainy 2024	Loamy Sand	8.10	669.25	23.83	30.28
E5-WS24	Rainy 2024	Sandy loam	8.07	469.32	22.84	29.93
E6-WS24	Rainy 2024	Sandy loam	7.92	889.42	22.90	29.54
E7-DS25	Dry 2025	Loamy Sand	8.53	67.12	18.11	32.62
E8-DS25	Dry 2025	Clay	8.59	81.48	19.00	33.26
E9-DS25	Dry 2025	Loamy Clay	8.55	67.30	18.30	32.82
E10-WS25	Rainy 2025	Sandy loam	7.38	924.65	22.62	29.65
E11-WS25	Rainy 2025	Loamy Sand	7.61	807.08	24.08	30.82

* Environment code: (E1-DS24) Phu Khiao District, Chaiyaphum Province, in dry season 2024 (16°18′14.40″ N 102°11′07.23″ E); (E2-DS24) Chum Phae District, Khon Kaen Province, in dry season 2024 (16°27′35.36″ N 102°17′49.09″ E); (E3-DS24) KKU, Khon Kaen Province, in dry sea-son 2024 (16°28′20.84″ N 102°48′35.39″ E); (E4-WS24) KKU, Khon Kaen Province, in wet season 2024 (16°28′23.05″ N 102°48′32.11″ E); (E5-WS24) Ban Thaen District, Chaiyaphum Province, in wet season 2024 (16°25′14.62″ N 102°18′13.48″ E); (E6-WS24) Phu Khiao District, Chaiyaphum Province, in wet season 2024 (16°18′08.97″ N 102°11′08.33″ E); (E7-DS25) KKU, Khon Kaen Province, in dry season 2025 (16°28′22.92″ N 102°48′37.40″ E); (E8-DS25) Chum Phae District, Khon Kaen Province, in dry season 2025 (16°27′31.72″ N 102°16′51.46″ E); (E9-DS25) Ban Thaen District, Chaiyaphum Province in wet season 2025 (16°26′28.15″ N 102°16′51.93″ E); (E10-WS25) Khon Kaen Province, in wet season 2025 (16°27′31.72″ N 102°16′51.46″ E); (E11-WS25) Chum Phae District, Khon Kaen Province, in wet season 2025 (16°40′52.77″ N 102°00′45.08″ E).

**Table 9:** Expected Mean Square (EMS) from combined analysis in mixed-model terms across six genotypes (Fixed) and eleven environments (Random).

Source of Variation	df	MS	EMS	F-Test
E	e − 1	MS_E_	σ^2^ + gσ^2^_R(E)_ + rgσ^2^_E_	MS_E_/MS_R(E)_
Reps./E	e(r − 1)	MS_R(E)_	σ^2^ + gσ^2^_R(E)_	
G	(g − 1)	MS_G_	σ^2^ + rσ^2^_GE_ + re*k*_G_	MS_G_/MS_GE_
G × E	(g − 1)(e − 1)	MS_GE_	σ^2^ + rσ^2^_GE_	MS_G__E_/MS_e_
Error	e(g − 1)(r − 1)	MS_e_	σ^2^	

E = environment, G = genotype, G × E = genotype by environment interaction, Reps./E = replication within environment, r = number of replications, g = number of genotypes, e = number of environments, df = degree of freedom, MS = mean square, σ = variance of random effect and *k* = variance of fixed effect.

## Data Availability

The data presented in this study are available upon request from the corresponding author.

## Supplementary Materials

The following supporting information can be downloaded at: https://www.mdpi.com/article/10.3390/plants15182820/s1. Supplementary Figure S1. Temperature, total photolytically active radiation (PAR), relative humidity (RH) and rainfall during yield trial recorded at: (A) Phu Khiao District, Chaiyaphum Province in dry season 2024; (B) Chum Phae District, Khon Kaen Province in dry season 2024; (C) KKU, Khon Kaen Province in dry season 2024; (D) Ban Thaen District, Chaiyaphum Province in wet season 2024; (E) Phu Khiao District, Chaiyaphum Province in wet season 2024 and (F) KKU, Khon Kaen Province in wet season 2024. Supplementary Figure S2. Temperature, total photolytically active radiation (PAR), relative humidity (RH) and rainfall during yield trial recorded at: (A) KKU, Khon Kaen Province in dry season 2025; (B) Chum Phae District, Khon Kaen Province in dry season 2025; (C) Ban Thaen District, Chaiyaphum Province in wet season 2025; (D) KKU, Khon Kaen Province in wet season 2025; (E) Chum Phae District, Khon Kaen Province in wet season 2025.
