# Supplementary material for: Yield Stability and Adaptability of Black Soybean Line BSCM41 Support Bi-Directional Seed Rotation System in Northeast Thailand
Source: Plants (Basel). 2026 Sep 14;15(18):2820. doi: 10.3390/plants15182820 (PMC13610238; doi:10.3390/plants15182820)
Supplement: Supplementary file 1 [file plants-15-02820-s001.zip › plants-4504422-supplementary.pdf]

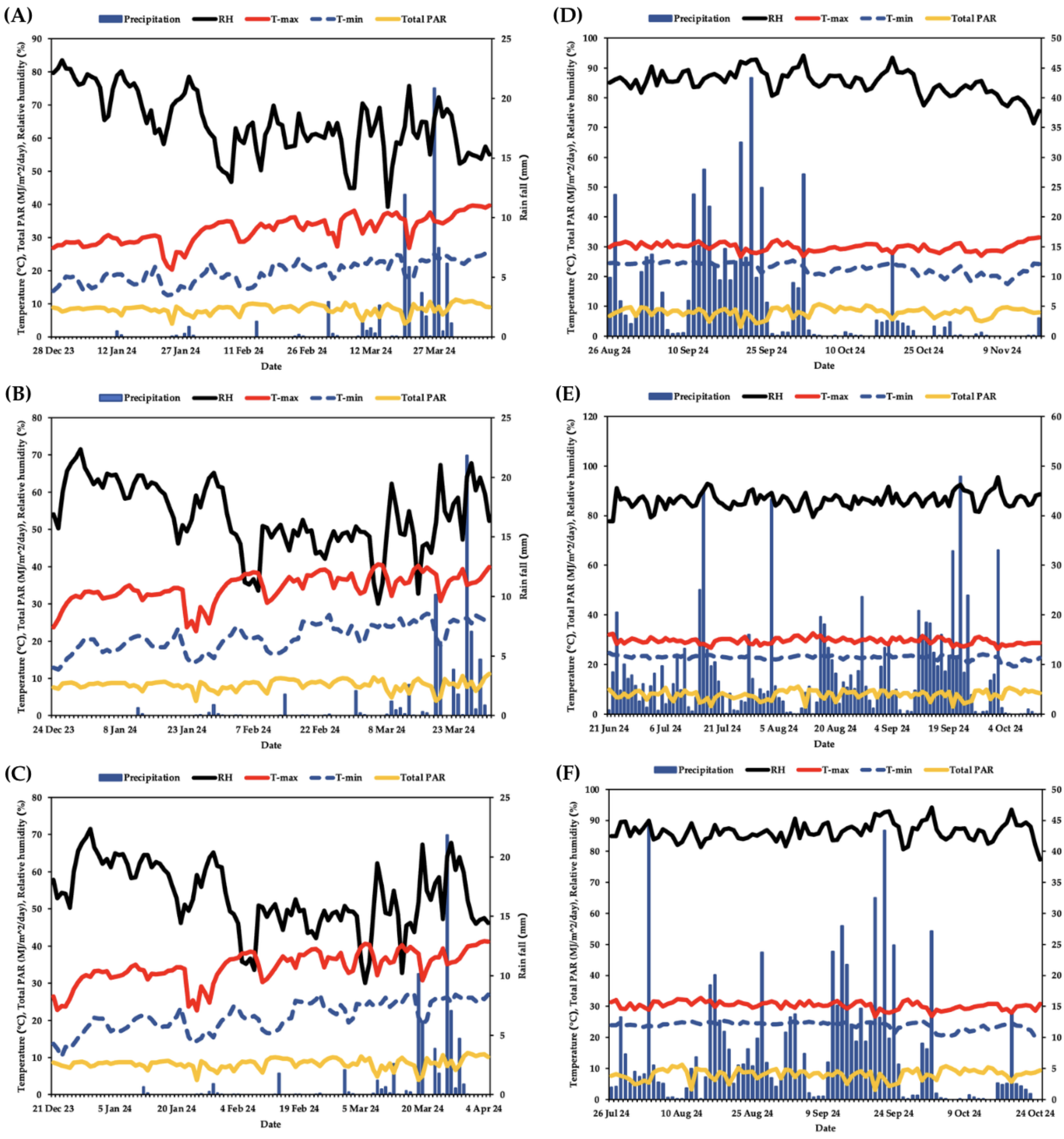

Supplementary Figure S1. Temperature, total photolytically active radiation (PAR), relative humidity (RH) and rainfall during yield trial recorded at: (A) Phu Khiao District, Chaiyaphum Province in dry season 2024; (B) Chum Phae District, Khon Kaen Province in dry season 2024; (C) KKU, Khon Kaen Province in dry season 2024; (D) Ban Thaen District, Chaiyaphum Province in wet season 2024; (E) Phu Khiao District, Chaiyaphum Province in wet season 2024 and (F) KKU, Khon Kaen Province in wet season 2024.

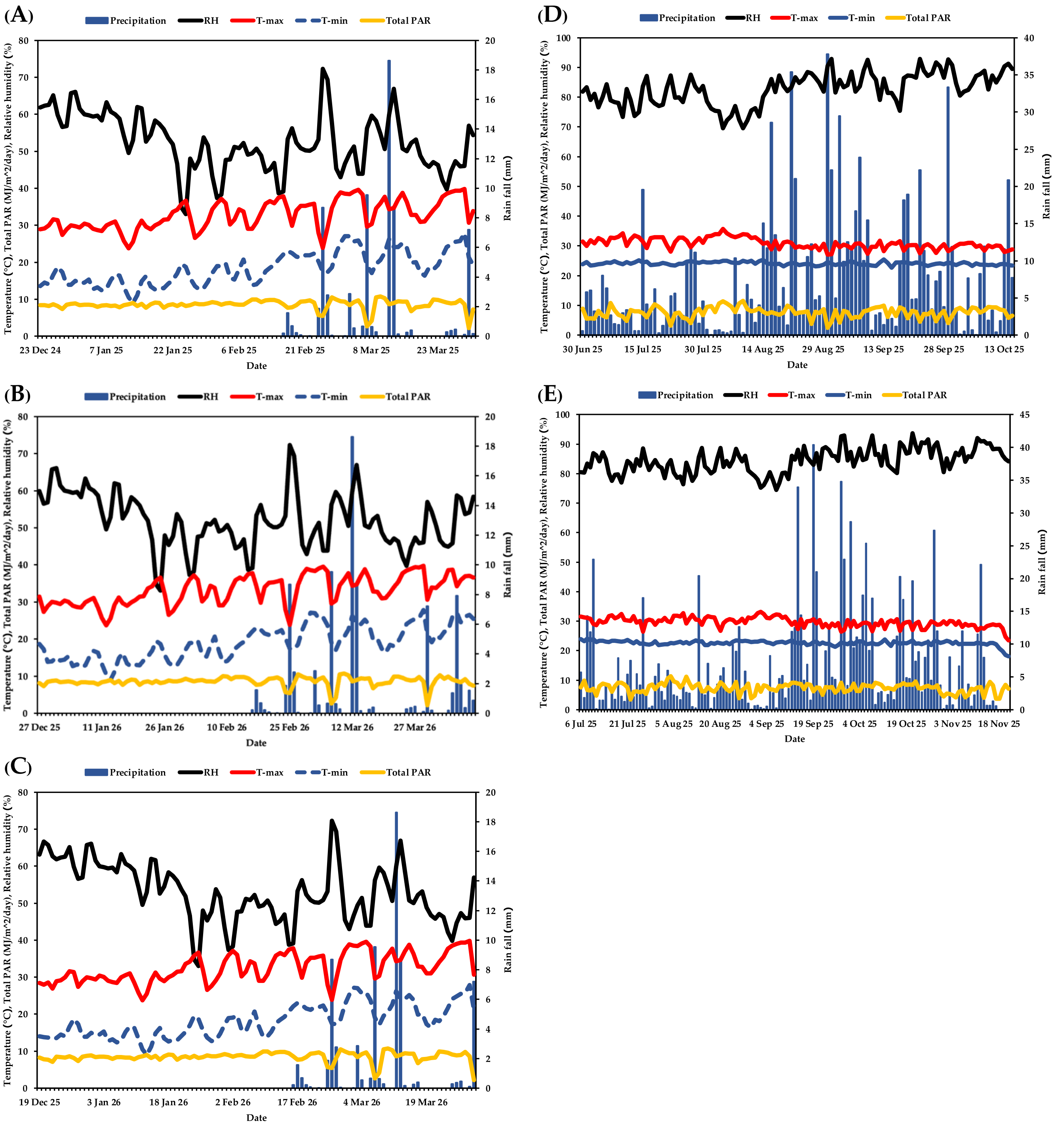

Supplementary Figure S2. Temperature, total photolytically active radiation (PAR), relative humidity (RH) and rainfall during yield trial recorded at: (A) KKU, Khon Kaen Province in dry season 2025; (B) Chum Phae District, Khon Kaen Province in dry season 2025; (C) Ban Thaen District, Chaiyaphum Province in wet season 2025; (D) KKU, Khon Kaen Province in wet season 2025; (E) Chum Phae District, Khon Kaen Province in wet season 2025.
